# Intra-Amniotic Administration (*Gallus gallus*) of *Cicer arietinum* and *Lens culinaris* Prebiotics Extracts and Duck Egg White Peptides Affects Calcium Status and Intestinal Functionality

**DOI:** 10.3390/nu9070785

**Published:** 2017-07-21

**Authors:** Tao Hou, Nikolai Kolba, Raymond P. Glahn, Elad Tako

**Affiliations:** 1 College of Food Science and Technology, Huazhong Agricultural University, Wuhan 430070, China; mrhoutao@webmail.hzau.edu.cn; 2 Department of Food Science and Technology, Cornell University, Ithaca, NY 14850, USA; 3 USDA-ARS, Robert W. Holley Center for Agriculture and Health, Cornell University, Ithaca, NY 14850, USA; nk598@cornell.edu (N.K.); rpg3@cornell.edu (R.P.G.)

**Keywords:** prebiotics, chickpea, lentil, duck egg white peptides, calcium, brush border membrane, microbial populations

## Abstract

Calcium (Ca) is one of the most abundant inorganic elements in the human body and has many important physiological roles. Prebiotics and bioactive peptides are two important substances used to promote calcium uptake. However, the difference in mechanisms of the calcium uptake from these two supplements is not clear. By using the *Gallus gallus* model and the intra-amniotic administration procedure, the aim of this study was to investigate whether Ca status, intestinal functionality, and health-promoting bacterial populations were affected by prebiotics extracted from chickpea and lentil, and duck egg white peptides (DPs). Eleven groups (non-injected; 18 MΩ H_2_O; 4 mmol/L CaCl_2_; 50 mg/mL chickpea + 4 mmol/L CaCl_2_; 50 mg/mL lentil + 4 mmol/L CaCl_2_; 40 mg/mL DPs + 4 mmol/L CaCl_2_; 5 mg/mL Val-Ser-Glu-Glu (VSEE) + 4 mmol/L CaCl_2_; 50 mg/mL chickpea; 50 mg/mL lentil; 40 mg/mL DPs; 5 mg/mL VSEE) were utilized. Upon hatch, blood, cecum, small intestine, liver and bone were collected for assessment of serum bone alkaline phosphate level (BALP), the relative abundance of intestinal microflora, expression of Ca-related genes, brush border membrane (BBM) functional genes, and liver and bone mineral levels, respectively. The BALP level increased in the presence of lentil, DPs and VSEE (*p* < 0.05). The relative abundance of probiotics increased significantly (*p* < 0.05) by VSEE + Ca and chickpea. The expression of CalbindinD9k (Ca transporter) increased (*p* < 0.05) in Ca, chickpea + Ca and lentil + Ca groups. In addition, the brush border membrane functionality genes expressions increased (*p* < 0.05) by the chickpea or lentil extracts. Prebiotics and DPs beneficially affected the intestinal microflora and duodenal villus surface area. This research expands the understanding of the prebiotics’ properties of chickpea and lentil extracts, and peptides’ effects on calcium metabolism and gut health.

## 1. Introduction

Calcium (Ca) homeostasis is vital to the human body and current Ca intake is below the recommended 1300 mg/day for adolescents in the USA [1]. Increasing calcium uptake would be an effective strategy for avoiding calcium deficiency [2]. However, if calcium intake remains inadequate, the improvement of calcium bioavailability becomes an important way to keep calcium homeostasis [3]. The search for functional foods that promote calcium uptake and bone formation has stimulated studies on alternative foods with this potential [4,5]. Among these foods, two kinds of substances are the focus of the current research: prebiotics [6,7] and peptides [4,8].

Prebiotics are non-digestible food ingredients that beneficially affect gut health [9,10] and improve mineral absorption [2,6]. Roberfroid et al. (2002) found that increased bone density was observed in the conventional rat fed a diet supplemented with 5% or 10% of inulin [11]. It was shown that supplementing diets with the GOS (gluco oligo saccharides)/FOS (fructo oligo saccharides) mixture increased bone mineralization, density and structure due to the increase in Ca, P and Mg absorption in growing rats [2]. Yacon flour in combination with *Bifidobacterium longum* increased the concentration of minerals in bones [12] and moderate daily intake of soluble maize fiber increased short-term Ca absorption in adolescents consuming less than the recommended amounts of Ca [13]. The increase of calcium bioavailability has been attributed to increased solubility of minerals due to increased bacterial production of short chain fatty acids (SCFA) via increased supply with substrate [14]; enlargement of the absorption surface by promoting proliferation of enterocytes mediated by bacterial fermentation products, predominantly lactate and butyrate [15] and increased expression of calcium binding proteins [16].

Apart from prebiotics, many calcium-chelating peptides, which can promote calcium uptake and bioavailability, have been discovered and characterized in recent years. For example, casein phosphopeptides (CPPs), phosphorylated peptides derived from milk, can be chelated with calcium to form soluble and stable complexes that were effective in promoting calcium absorption [17]. In addition, whey [18], Alaska pollock backbone and skin [19], tilapia fish muscle and scales [20], soy [21], shrimp [22] and chlorella [23] also have been proved to promote calcium uptake by chelating calcium via the carboxyl groups of glutamine (Glu) and aspartic acid (Asp) residues. Apart from the binding capacity, peptides contribute calcium uptake by the direct interaction with the plasma membrane but it did not influence ion channels in differentiated human colon colorectal adenocarcinoma (HT-29) cells [24]. More recently, researchers found that CPPs modulated calcium uptake through their interaction with a calcium channel in the plasma membrane. In human epithelial colorectal adenocarcinoma (Caco-2) cells, calcium absorption occurs via the Transient receptor potential vanilloid 6 (TRPV6) channel [25]. Instead, in HT-29 cells, it involves the voltage-operated L-type calcium channels, in the form of the Cav1.3 subunit [26].

Previously, the *Gallus gallus* model was shown to exhibit the appropriate responses to Fe deficiency and that it can serve as a model for human Fe bioavailability [27]. In the current study, the effect of prebiotics (extracted from chickpea and lentil) and duck egg white peptides on the promotion of calcium uptake was studied in vivo by utilizing the intra-amniotic administration model (*Gallus gallus*) [28]. As is the case in humans and the vast majority of animals, *Gallus gallus* harbors a complex and dynamic gut microbiota, heavily influenced by host genetics, environment, and diet [29]. There is considerable similarity at the phylum level between the gut microbiota of broilers (*Gallus gallus*) and humans, with Bacteroidetes, Firmicutes, Proteobacteria, and Actinobacteria representing the four dominant bacterial phyla in both [30]. Due to its rapid maturation and well–characterized phenotype during mineral deficiency, *Gallus gallus* has been used extensively as a model of human nutrition, especially as it pertains to assessing physiological outcomes of low dietary Fe and Zn [31,32,33].

Chickpea and lentil are staple food crops that are high in fibers [34,35]. It was previously suggested that chickpea and lentil prebiotics have the potential to modulate the intestinal microbial composition to promote intestinal health in humans [36,37]. Duck egg white peptides (DPs) have been proven to have the function on promotion of calcium uptake in calcium-deficient diets rat model [38] and phytic acid-induced calcium restriction mice model [39]. Thus, the primary objective of this study is to assess and compare the effects of intra-amniotic prebiotics and peptides administration on Ca status, Ca metabolism-related genes (TRPV6, CalbindinD9k, CalbindinD28k, Plasma membrane Ca^2+^ ATPase (PMCA1b), Secreted Phosphoprotein 1 (SPP1), and Sodium calcium exchanger 1 (NCX1)) and brush border membrane development and functionality such as the relative expressions of aminopeptidase (AP) and sucrase isomaltase (SI) in vivo. A secondary objective is to evaluate the effects of the intra-amniotic administration of these prebiotics and DPs on intestinal bacterial populations by measuring the relative abundances of probiotic health-promoting populations bacteria such as *Bifidobacterium* and *Lactobacillus* versus those of potentially pathogenic bacteria such as *E. coli* and *Clostridium*. We hypothesized that prebiotics and peptides could both improve calcium status, brush border membrane (BBM) functionality and intestinal bacterial populations. 

## 2. Materials and Methods

### 2.1. Extraction of Prebiotics

As was previously described [40], the milled whole seed chickpea and lentil samples were dissolved in distilled water (50 g/L) (60 °C, 90 min) and then centrifuged at 3000 rpm for 20 min to remove particulate matter and then centrifuged at 3000 rpm for 10 min at 4 °C. The supernatant was collected and dialysed (MWCO 12–14 kDa) exhaustively against distilled water for 48 h. At last, the dialysate was collected and then lyophilized to yield a fine off-white powder.

### 2.2. Preparation of Desalted Duck Egg White Peptides (DPs)

DPs with a molecular weight of less than 5 kDa were prepared from salted duck egg white as was previously described [30]. Briefly, salted duck egg white was desalted by the electrodialysis equipment, followed by denaturation for 30 min in boiling water and cooling to 50 °C. The pH was adjusted to 6.5 prior to the addition of protamex (Enzyme:Substrate = 1:25) and maintained at 6.5. After 3.5 h, the hydrolyzed solution was bathed in boiling water for 10 min to inactivate the enzyme. The degree of hydrolysis is 21.97%. Finally, the mixture was centrifuged at 3000 rpm for 10 min, and the supernatant was filtered through a hollow fiber membrane with a molecular weight cutoff of 5 kDa (PLCC, Millipore, Billerica, MA, USA). The filtrate was lyophilized and defined as DPs.

### 2.3. Animals and Design

Cornish-cross fertile broiler eggs (*n* = 110) were obtained from a commercial hatchery (Moyer’s chicks, Quakertown, PA, USA). The eggs were incubated under optimal conditions at the Cornell University Animal Science poultry farm incubator. All animal protocols were approved by Cornell University Institutional Animal Care and Use committee (ethic approval code: 2007-0129). Prebiotics, DPs and Val-Ser-Glu-Glu (VSEE) in powder form were separately diluted in 18 MΩ H_2_O to determine the concentrations necessary to maintain an osmolarity value (Osm) of less than 320 Osm to ensure that the chicken embryos would not be dehydrated upon injection of the solution. At 17 day of embryonic incubation, eggs containing viable embryos were weighed and divided into 11 groups (*n* = 10). All treatment groups were assigned eggs of similar weight frequency distribution. Each group was then injected with the specified solution (1 mL per egg) with a 21-gauge needle into the amniotic fluid, which was identified by candling. The 11 groups were assigned as follows: (1) non-injected; (2) 18 MΩ H_2_O; (3) 4 mmol/L CaCl_2_; (4) 50 mg/mL chickpea + 4 mmol/L CaCl_2_; (5) 50 mg/mL lentil + 4 mmol/L CaCl_2_; (6) 40 mg/mL DPs + 4 mmol/L CaCl_2_; (7) 5 mg/mL VSEE + 4 mmol/L CaCl_2_; (8) 50 mg/mL chickpea; (9) 50 mg/mL lentil; (10) 40 mg/mL DPs; and (11) 5 mg/mL VSEE. After all the eggs were injected, the injection holes were sealed with cellophane tape and the eggs placed in hatching baskets such that each treatment was equally represented at each incubator location. Immediately after hatch (21 days) and from each treatment group, chicks were euthanized by CO_2_ exposure and their small intestine, blood, cecum, liver and bone were collected.

### 2.4. Serum Bone Alkaline Phosphatase and Liver and Bone Mineral Analysis

Serum bone alkaline phosphatase (BALP) was measured by ELISA kits (MyBioSource, Vancouver, BC, Canada). The liver and bone Ca, Mg, Zn, and Fe concentrations were determined by an inductively-coupled argon-plasma/atomic emission spectrophotometer (ICAP 61E Thermal Jarrell Ash Trace Analyzer, Jarrell Ash Co., Franklin, MA, USA).

### 2.5. Isolation of Total RNA from Chicken Duodenum

Total RNA was extracted from 30 mg of the proximal duodenal tissue (*n* = 88) using Qiagen RNeasy Mini Kit (RNeasy Mini Kit, Qiagen Inc., Valencia, CA, USA) according to the manufacturer’s protocol. Total RNA was eluted in 50 μL of RNase free water. All steps were carried out under RNase free conditions. RNA was quantified by absorbance at A 260/280. RNA was stored at −80 °C until used.

### 2.6. Real Time Polymerase Chain Reaction (RT-PCR)

To create the cDNA, a 20 μL reverse transcriptase (RT) reaction was completed in a BioRad C1000 touch thermocycler using the Improm-II Reverse Transcriptase Kit (Catalog #A1250; Promega, Madison, WI, USA). The first step consisted of 1 μg of total RNA template, 10 μM of random hexamer primers, and 2 mM of oligo-dT primers. The RT protocol was to anneal primers to RNA at 94 °C for 5 min, copy the first strand for 60 min at 42 °C (optimum temperature for the enzyme), then heat inactivate at 70 °C for 15 min and hold at 4 °C until ready to analyze by Nanodrop (Waltham, MA, USA). The concentration of cDNA obtained was determined by measuring the absorbance at 260 nm and 280 nm using an extinction coefficient of 33 (for single stranded DNA). Genomic DNA contamination was assessed by a real-time RT-PCR assay for the reference genes samples.

### 2.7. Isolation of Chicken Intestinal CalbindinD9k Gene Fragment

Primers were designed corresponding to nucleotides 115–137 (5′-CCT GCA GAA ATG AAG AGC ATT TT-3′) and 266–286 (5′-CAA AAA TAT GCA GCC AAG GAA GGC GA-3′) of the previously published mouse calbindinD9k sequence [41]. For each PCR, cDNA (2 μg) was used for each 10 μL reaction together with 2× SsoAdvanced Universal SYBR Green Supermix (Kit #1725271; BioRad, Hercules, CA, USA) which included buffer, Taq DNA polymerase, dNTPs and SYBR green dye. The reaction was performed with primer S-100F: 5′-CCTGCAGAAATGAAGAGCATTTT-3′ and S-100R: 5′-CTCCATCGCCATTCTTATCCA-3′ [33]. PCR was carried out according to the following parameters: 50 °C for 2 min, initial denaturing at 95 °C for 2 min, 40 cycles of denaturing at 95 °C for 15 s, 65 °C for 15 s and elongating at 72 °C for one minute. We electrophoresed the resulting PCR products on a 2%-agarose gel, stained the gel with ethidium bromide, and visualized it under UV light. PCR-positive products were purified of primer dimers and other non-specific amplification by-products using ExoSAP-IT for PCR Product Clean-up (Affymetrix, Cleveland, OH, USA) prior to sequencing. We sequenced the products using BigDye^®^Terminator v3.1 Cycle Sequencing Kits (Applied Biosystems, Foster City, CA, USA) and ABI Automated 3430xl DNA Analyzer (Applied Biosystems) and analyzed them with Sequencing Analysis ver. 5.2 (Applied Biosystems). Sequences of CalbindinD9k were aligned with those from related organisms obtained from Gen Bank using a basic alignment-search tool (BLAST; National Center for Biotechnology Information, Bethesda, MD, USA). Sequence alignments were performed for all samples. The ClustalW program was used for sequence alignment. We obtained known CalbindinD9k sequences from GenBank for sequence alignment.

### 2.8. Primer Design

The calcium and iron primers used in the real-time PCR were designed based on 12 gene sequences from Genbank database, using Real-Time Primer Design Tool software (IDT DNA, Coralvilla, IA, USA). The sequences and the description of the primers used in this work are summarized in Table 1. The amplicon length was limited to 90 to 150 bp. The length of the primers was 17–25-mer and the GC content was between 41% and 55%. The specificity of the primers was tested by performing a BLAST search against the genomic National Center for Biotechnology Information (NCBI) database.

The *Gallus gallus* primer 18S rRNA was designed as a reference gene. Results obtained from the qPCR system were used to normalize those obtained from the specific systems as described below. 

### 2.9. Real-Time qPCR Design

As was previously described [42], cDNA was used for each 10 μL reaction together with 2× BioRad SSO Advnaced Universal SYBR Green Supermix (Cat #1725274, Hercules, CA, USA) which included buffer, Taq DNA polymerase, dNTPs and SYBR green dye. Specific primers (forward and reverse (Table 1) and cDNA or water (for no template control) were added to each PCR reaction. The specific primers used can be seen in Table 1. For each gene, the optimal MgCl_2_ concentration produced the amplification plot with the lowest cycle product (Cp), the highest fluorescence intensity and the steepest amplification slope. Master mix (8 µL) was pipetted into the 96-well plate and 2 µL cDNA was added as PCR template. Each run contained 7 standard curve points in duplicate. A no template control of nuclease-free water was included to exclude DNA contamination in the PCR mix. The double stranded DNA was amplified in the Bio-Rad CFX96 Touch (Hercules, CA, USA) using the following PCR conditions: initial denaturing at 95 °C for 30 s, 40 cycles of denaturing at 95 °C for 15 s, various annealing temperatures according to Integrated DNA Technologies (IDT) for 30 s and elongating at 60 °C for 30 s. The data on the expression levels of the genes were obtained as Cp values based on the “second derivative maximum” (=automated method) as computed by the software. For each of the 12 genes, the reactions were run in duplicate. All assays were quantified by including a standard curve in the real-time qPCR analysis. The next four points of the standard curve were prepared by a 1:10 dilution. Each point of the standard curve was included in duplicate. A graph of Cp vs. log 10 concentrations was produced by the software and the efficiencies were calculated as 10[1/slope]. The specificity of the amplified real-time RT-PCR products were verified by melting curve analysis (60–95 °C) after 40 cycles, which should result in a number of different specific products, each with a specific melting temperature. In addition, we electrophoresed the resulting PCR products on a 2%-agarose gel, stained the gel with ethidium bromide, and visualized it under UV light. PCR-positive products were purified of primer dimers and other non-specific amplification by-products using QIAquick Gel Kit (Qiagen Inc., Valencia, CA, USA) prior to sequencing. We sequenced the products using BigDye^®^Terminator v3.1 Cycle Sequencing Kits (Applied Biosystems, Foster City, CA, USA) and ABI Automated 3430xl DNA Analyzer (Applied Biosystems) and analyzed them with Sequencing Analysis ver. 5.2 (Applied Biosystems). We aligned sequences of CalbindinD9k with those from related organisms obtained from Gen Bank using a basic alignment-search tool (BLAST; National Center for Biotechnology Information, Bethesda, MD, USA). Sequence alignments were performed for all samples. We used the ClustalW program for sequence alignment. Real-time RT-PCR efficiency (E) values for the five genes were as follows: CalBindinD9K, 0.996; CalBindinD28K, 0.966; NCX1, 0.951; PMCA1b, 0.935; SPP1, 1.032; Alkaline Phosphatase (AKP), 1.288; AP, 1.015; Divalent metal transporter 1 (DMT1), 0.988; 18S Ribosomal subunit (18S rRNA), 0.934; Zinc transporter 4 (Zip4), 0.994; SI, 1.032; TRPV6, 1.015

### 2.10. Collection of Microbial Samples and Intestinal Contents DNA Isolation

The ceca were sterilely removed and treated as described previously [43]. The contents of the ceca were placed into a sterile 50 mL tube containing 9 mL of sterile PBS and homogenized by vortexing with glass beads (3 mm diameter) for 3 min. Debris was removed by centrifugation at 700× *g* for 1 min, and the supernatant was collected and centrifuged at 12,000× *g* for 5 min. The pellet was washed twice with PBS and stored at −20 °C until DNA extraction. For DNA purification, the pellet was re-suspended in 50 mM EDTA and treated with lysozyme (Sigma Aldrich CO., St. Louis, MO, USA; final concentration of 10 mg/mL) for 45 min at 37 °C. The bacterial genomic DNA was isolated using a Wizard Genomic DNA purification kit (Promega Corp., Madison, WI, USA).

### 2.11. Primers Design and PCR Amplification of Bacterial 16S rDNA

Primers for *Lactobacillus*, *Bifidobacterium*, *Clostridium* and *E. coli* were designed according to previously published data [44]. To evaluate the relative proportion of each examined bacteria, all products were expressed relative to the content of the universal primer product and proportions of each bacterial group are presented. PCR products were separated by electrophoresis on 2% agarose gel, stained with ethidium bromide, and quantified using the Quantity One 1-D analysis software (Bio-Rad, Hercules, CA, USA).

### 2.12. Morphological Examination

As was previously described [42], intestinal samples (duodenal region as the main intestinal Ca absorption site) at day of hatch from each treatment were fixed in fresh 4% (*v*/*v*) buffered formaldehyde, dehydrated, cleared, and embedded in paraffin. Serial sections were cut at 5 μm and placed on glass slides. Sections were deparaffinized in xylene, rehydrated in a graded alcohol series, stained with hematoxylin and eosin, and examined by light microscopy. Morphometric measurements of villus height, width and goblet cell diameter were performed with a light microscope using EPIX XCAP software (Olympus, Waltham, MA, USA). Villus surface area was calculated from villus height and width at half height.

### 2.13. Statistical Analysis 

Experimental data were presented as mean values ± SEM. The mean values were compared by Duncan’s multiple range test at *p* < 0.05 using SAS software ver.8.1 (SAS, Carey, NC, USA).

## 3. Results

### 3.1. Isolation and Sequencing of Partial Chicken Intestinal CalbindinD9k cDNA

**Figure 1:**
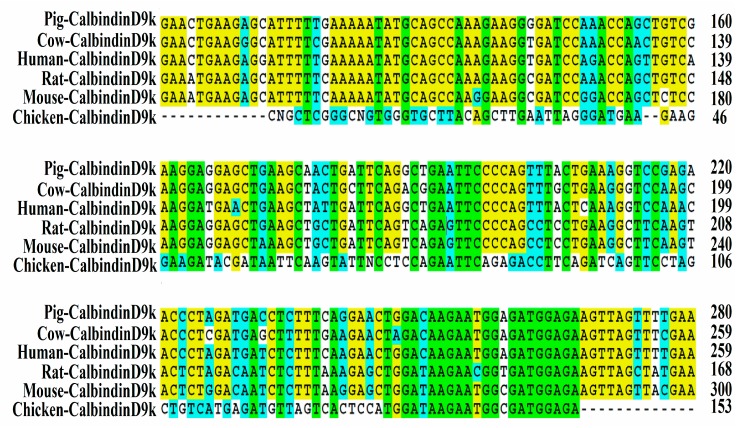
Predicted partial amino acid sequences of the chicken intestinal CalbindinD9k (Ca transporter D9k) exporter. The alignment of predicted amino acid sequences of chicken intestinal CalbindinD9k with pig CalbindinD9k (GI397265), cow CalbindinD9k (GI281658), human CalbindinD9k (GI65795), mouse CalbindinD9k (GI12309) and rat CalbindinD9k (GI24249) is shown. Homologous residues are shaded.

As shown in Figure 1, a 153-bp fragment of the chicken intestinal CalbindinD9k gene was isolated by reverse transcriptase-PCR and subjected to sequence analysis. It exhibited 24% homology to *Rattus novergicus* and 22% homology to *Sus scrofa* and *Homo sapiens* intestinal CalbindingD9K genes. The cDNA sequence of the chicken intestinal CalbindinD9k was entered into the BioSample nucleotide sequence database under accession number SAMN06269460.

### 3.2. Body and Cecum Weight

**Figure 2:**
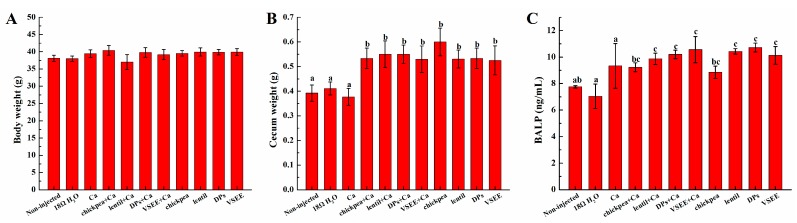
The effect of chickpea prebiotics, lentil prebiotics, Desalted duck egg white peptides (DPs) and Val-Ser-Glu-Glu (VSEE) on the: (**A**) body weight; (**B**) cecum weight; and (**C**) serum bone alkaline phosphate. Values are means ± SEM. ^a,b,c^ within a column, means without a common letter are significantly different, *p* < 0.05 (Duncan’s multiple range test at *p* < 0.05.

As shown in Figure 2A, no significant difference was observed in body weight between treatment groups (*p* > 0.05). However, the cecum weight in treatment groups except for Ca group were significantly higher compared to the non-injected and 18 MΩ H_2_O groups (*p* < 0.05, Figure 2B).

### 3.3. Serum Bone Alkaline Phosphate (BALP) Content

The BALP levels in lentil + Ca, DPs + Ca, VSEE + Ca, lentil, DPs and VSEE groups were all significantly higher than the non-injected, 18 MΩ H_2_O and Ca groups (*p* < 0.05, Figure 2C). The BALP content in Ca group increased but no significant difference was observed compared to the non-injected and 18 MΩ H_2_O groups (*p* > 0.05).

### 3.4. Liver and Bone Mineral Content

As shown in Table 2, no significant differences were observed in bone and liver Ca, Mg, Fe, and Zn content (*p* > 0.05).

### 3.5. Intestinal Content Bacterial Genera- and Species-Level Analysis

**Figure 3:**
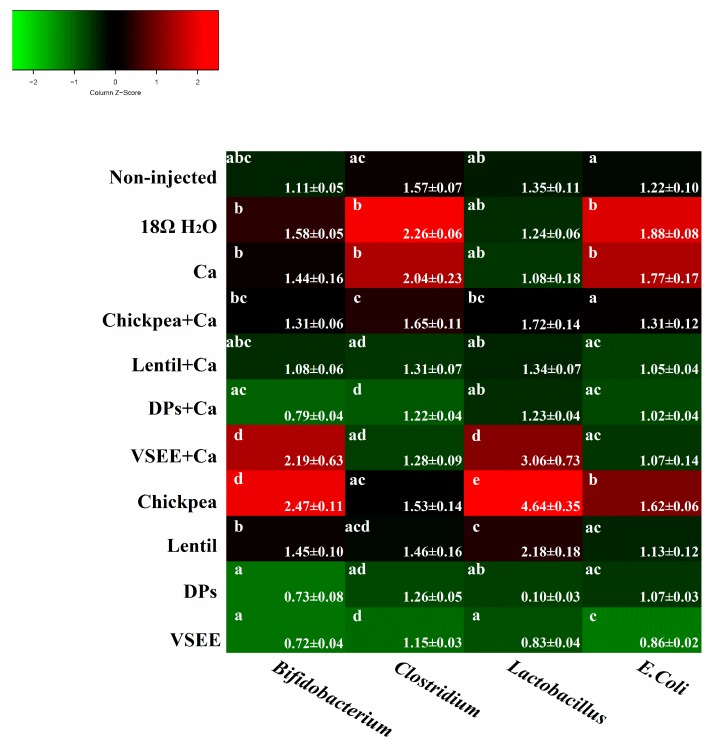
Genera and species-level bacterial populations (AU) from cecal contents measured on the day of hatch. Values are means ± SEM, *n* = 8. ^a–d^ Per bacterial category, treatment groups not indicated by the same letter are significantly different (*p* < 0.05).

As shown in Figure 3, the relative abundance of both Bifidobacterium and Lactobacillus genera, increased (*p* < 0.05) in the presence of VSEE + Ca and chickpea, relative to the non-injected group. The relative abundance of Bifidobacterium in other groups did not increase or decrease significantly compared to the non-injected, 18 MΩ H_2_O and Ca groups. The Lactobacillus content also increased significantly (*p* < 0.05) in lentil group and other five treatment groups did not show any significant increase or decrease compared to non-injected, 18 MΩ H_2_O and Ca groups. The relative abundance of *E. coli* significantly increased (*p* < 0.05) in the 18 MΩ H_2_O, Ca and chickpea groups and significantly decreased (*p* < 0.05) in VSEE group compared to the non-injected group. Clostridium’s relative abundance significantly (*p* < 0.05) increased in the 18 MΩ H_2_O and Ca groups and decreased in DPs + Ca and VSEE groups compared to the non-injected group. These results indicated that chickpea and lentil could improve gut health by promoting the survival of probiotics. However, this effect was not observed in the presence of DPs, DPs + Ca and VSEE. Prebiotics from chickpea and lentil and peptides from egg white could limit the presence of potentially pathogenic bacterial populations.

### 3.6. Morphometric Measurements

**Figure 4:**
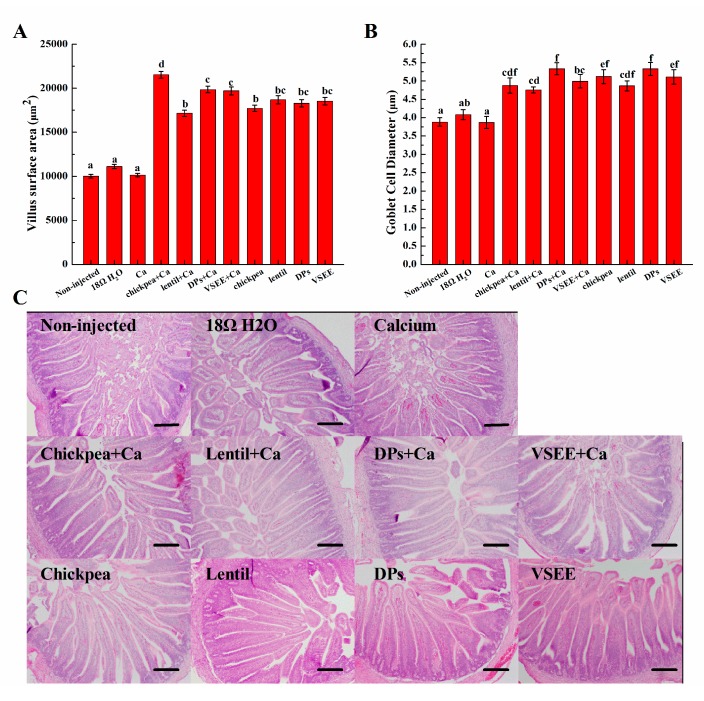
Effect of intra-amniotic administration of experimental solutions on: (**A**) the duodenal small intestinal villus surface area; and (**B**) goblet cells diameters. A representation of intestinal morphology from five dependent experiments in each group is shown (**C**). Values are means ± SEM, *n* = 5. Standard errors are represented by vertical bars. ^a–f^ Treatments groups not indicated by the same letter are significantly different (*p* < 0.05). Bar = 100 µm.

The villus surface areas and diameter of goblet cells significantly (*p* < 0.05) increased in all treatment groups except for the Ca group (Figure 4). This serves as a mechanical measurement of brush border membrane absorptive ability and improvement in brush border membrane functionality and gut health. It indicated that the supplement of prebiotics from chickpea, lentil and peptides from egg white could lead to a proliferation of enterocytes.

### 3.7. Calcium Metabolism Genes

**Figure 5:**
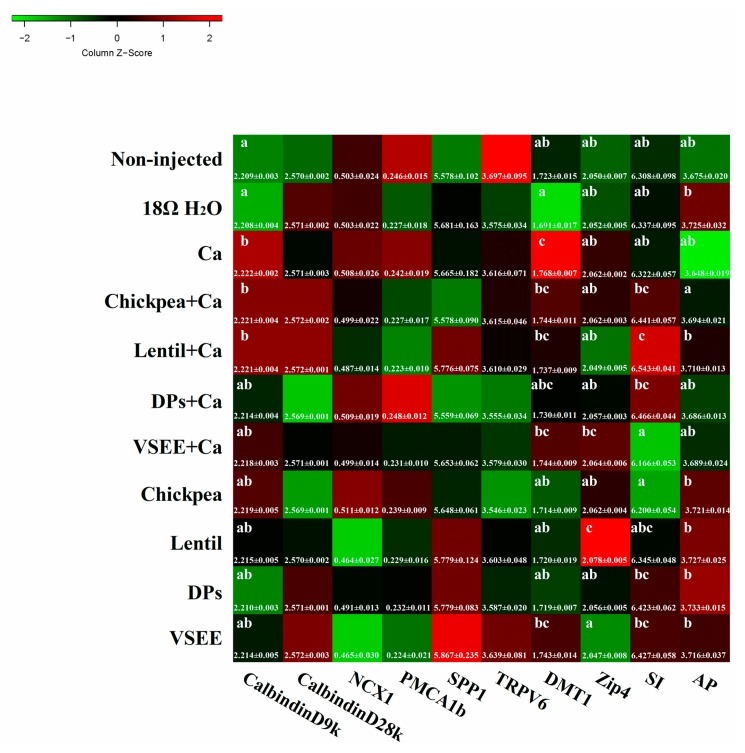
Effect of intra-amniotic administration of experimental solutions on the intestinal gene expression. Values are means ± SEM, *n* = 5. ^a–c^ Per gene, treatments groups not indicated by the same letter are significantly different (*p* < 0.05). AP, Amino peptidase; SI, Sucrose isomaltase; Zip4, Zinc transporter 4; DMT1, Divalent metal transporter 1; TRPV6, Transient receptor potential vanilloid 6 calcium channel; SPP1, Secreted Phosphoprotein 1; PMCA1b, Plasma membrane Ca^2+^ ATPase; NCX1, Sodium calcium exchanger 1; CalBindinD28k, Ca transporter D28k; CalBindinD9k, Ca transporter D9k.

It is widely accepted that there are two distinct pathways involved in calcium absorption across epithelia: one transcellular, the other paracellular [45]. Transcellular calcium absorption is generally envisaged as a three-step process: (1) the entrance of Ca^2+^ across the BBM of enterocytes through epithelial Ca^2+^ channels TRPV6, TRPV5, and Cav1.3; (2) Ca^2+^ movement to the basolateral membranes by binding proteins such as CalbindinD9k or CalbindinD28k; and (3) Ca^2+^ extrusion into the blood by the plasma membrane Ca^2+^ ATPase (PMCA1b) or sodium calcium exchanger (NCX1) [46]. Phosphoprotein 1 (SPP1) plays some functional roles in biomineralizationa and bone modeling [47,48]. As shown in Figure 5, there were no significant differences of TRPV6, CalbindinD28k, PMCA1b, NCX1 and SPP1 genes expressions (all are Ca^2+^ transporters) observed in the treatment groups compared to the non-injected group (*p* > 0.05). The gene expression of CalbindinD9k, which was a calcium transporter protein, increased significantly in Ca, chickpea + Ca, lentil + Ca groups compared to the non-injected group (*p* < 0.05). This indicated that DPs and VSEE might facilitate calcium transport by acting as calcium carriers.

### 3.8. BBM Functional Genes

The gene expression of DMT1, which is the common receptor of divalent metal ions, increased significantly in the Ca group compared to other groups (*p* < 0.05, Figure 5). This indicated that DMT1 might also be involved in calcium BBM transport. Zip 4 plays an important role in the transport of zinc. In current study, the relative expression of Zip4 was significantly up-regulated by lentil (*p* < 0.05). Sucrase Isomaltase (SI) and aminopeptidase (AP) are used as two biomarkers for BBM absorptive ability in this study. The relative expression of SI was up-regluated by lentil + Ca (*p* < 0.05). There was no significant difference of AP gene expression observed in all treatment groups compared to the non-injected group (*p* > 0.05).

## 4. Discussion

The results of the present study demonstrated that intra-amniotic administration of prebiotics (chickpea and lentil) and DPs might improve Ca bioavailability in several pathways: (1) the promotion of gut beneficial bacterial populations levels and limitation of potentially pathogenic bacterial populations; (2) the enlargement of gut villus surface area and the improvement of intestinal functionality; and (3) DPs and VSEE may potentially act as calcium transporter.

The broiler chicken (*Gallus gallus*) has been widely used to assess dietary mineral bioavailability [28,40,49,50]. This animal model matures relatively quickly and is sensitive to dietary manipulation of various micronutrients such as zinc and iron [28,51]. Previously, the intra amniotic administration approach was used to demonstrate the potential effect of plant origin prebiotics such as wheat [40], inulin [28], raffinose and stachyose [42] on Fe bioavailability and gut functionality.

Other animal models are also used for in vivo assessment of calcium bioavailability [2,4,14]. For example, the growing rat model was employed by Choi et al. (2005) to examine effectiveness of phosvitin peptides in enhancing the bioavailability of calcium and its accumulation in bones [4]. Calcium-deficient rats were used in the evaluation of tilapia scale protein hydrolysate-calcium complex on the calcium uptake and bone formation [20]. In recent years, retinoic acid model of bone loss in rats were applied in several studies to access effects of various substances on the skeletal system [52,53]. The effect of different dosage of Nylestriol/Levonorgestrel on bone metabolism in female rats with retinoic acid-induced osteoporosis was studied and 0.15 mg/kg Nylestriol in combination with 0.015 mg/kg Levonorgestrel produced beneficial effects in bone metabolism [52]. Ovariectomized (OVX) rats are a known animal model for postmenopausal osteoporosis due to its bone loss and its sequelae, which resemble those found in postmenopausal women in one or more respects [54]. OVX has been employed to elucidate the beneficial effects of fish-bone peptides and bovine collagen peptides on Ca bioavailability, respectively [55,56]. As for exact mechanism studies, gene knockout studies might be more useful. Van Cromphaut et al. (2001) illustrate that transient receptor potential vanilloid 6 (TRPV6) plays a rate-limiting role in active calcium transport [45]. However, a series of studies have sometimes generated conflicting data on the effects on Ca^2+^ homeostasis and absorption in knockouts for vitamin D receptor, calbindinD9k, calbindinD28k and TRPV6 [46].

In this study and for the first time, the intra-amniotic administration model was used to evaluate the effect of prebiotics and peptides on the promotion of calcium uptake. There were significant increases (*p* < 0.05) of villus surface area and goblet cells diameter (Figure 4A,B) in all treatment groups except for Ca group compared to the non-injected group. This suggests that prebiotics (chickpea and lentil), DPs and VSEE increase the absorptive surface and therefore duodenal functionality by increasing the proliferation of intestinal enterocytes. This effect is in agreement with previous research indicating that prebiotics (such as fructooligosacchadides) were shown to promote the proliferation of enterocytes [16]. Casein phosphopeptides has also been reported to favor calcium absorption by acting as cellular bio-modulator and increase Caco-2 cells differentiation [57]. This is the first time that DPs and VSEE were shown to increase the villus surface area and goblet cells diameter by promoting the proliferation of enterocytes in vivo. Serum bone alkaline phosphate (BALP) is used for the early diagnosis of infantile rickets and it is the best indicator to evaluate human bone mineralization disorders [30]. When the precipitation of calcium salt is insufficient, the secretion of BALP increases 2-fold times than the healthy control [38]. As shown in Figure 2C, BALP level in all treatment groups (but the Ca group) were within the normal physiological range but significantly higher compared to the non- injected and 18 MΩ H_2_O groups. This indicates that the bone formation activity increases after supplement with calcium, prebiotics and peptides. The liver and bone mineral content in treatment groups did not show notably differences relative to the non-injected and 18 MΩ H_2_O groups. As was previously suggested, this could be caused by the relatively short exposure time [40].

In addition, *Bifidobacterium* and *Lactobacillus* increased significantly with VSEE + Ca, chickpea and lentil treatments compared to the non-injected, 18 MΩ H_2_O and Ca groups (*p* < 0.05, Figure 3). Our findings concur with previously published papers that demonstrate an increase in *Bifidobacterium* and *Lactobacillus* population in the presence of prebiotics that had also led to promote calcium bioavailability [3,58]. This is potentially due to the fermentation of prebiotics by colonic bacteria that promotes the production of unbranched SCFA such as acetic, propionic, butyric, and lactic acids [59]. It was previously reported that pH and peptide supply could alter bacterial populations and SCFA ratios within microbial communities and the butyrate concentration was higher in 0.6% peptide group compared to the 0.1% peptide group [60]. SCFA could release Ca from complex like phytic acid-Ca chelate by a lowering of the pH [40]. The present study provides the first evident that DPs and VSEE were shown to alter bacterial populations in vivo.

The relative abundance of *E-coli* and *Clostridium* were significantly (*p* < 0.05) higher in 18 MΩ H_2_O and Ca groups, and lower (*p* < 0.05) in VSEE group compared to the non-injected group (Figure 3). *Clostridium* is a potentially pathogenic genus and *E. coli* is either pathogenic or beneficial, depending on the strain [61]. This indicates that treatment with calcium alone might affect the balance of beneficial bacterial populations (*Bifidobacterium* and *Lactobacillus*) and other bacterial populations (*E-coli* and *Clostridium*), in which some species were demonstrated to be pathogenic. When prebiotics (chickpea and lentil) and DPs added, the gut environment maintained healthy and VSEE could reduce the pathogenic genus level. Therefore, it is reasonable to suspect that the intra-amniotic administration of prebiotics and peptides could maintain gut health and then improve Ca status by increasing intestinal transferring. 

Moreover, the cecum weight (Figure 2B) in all treatment except for Ca group was significantly higher (*p* < 0.05) than the non-injected and 18 MΩ H_2_O groups. This indicated that the cecal content that received intra-amniotic prebiotic and peptides solutions was greater than those that did not. This observation supported the hypothesis that the cecum-body weight ratio could be used as an indicator for a potential increase in cecal bacterial populations [42].

As mentioned before, there are two distinct pathways involved in calcium absorption across epithelia; one transcellular, and the other, paracellular [5]. In our previous studies, DPs and VSEE were shown to promote calcium uptake in Caco-2 cell monolayers; this may do so by acting as calcium carriers and interacting with the cell membrane to open a special Ca^2+^ channel, whereas the paracellular pathway may make only a minor contribution [62]. In vivo, DPs was found to regulate the proliferation and differentiation of enterocytes through the interaction with TRPV6 calcium channel [39]. Ferraretto et al. (2001) stated that CPPs enhanced calcium uptake in differentiated HT-29 cells via a direct interact with the plasma membrane but did not influence receptors or ion channels and CPPs might insert themselves into the plasma membrane and form their own calcium selective channels or act as calcium-carrier [24].

In the current study, DMT1 and calbindinD9k gene expressions significantly increased in the Ca group (*p* < 0.05, Figure 5). This indicates that DMT1 and calbindinD9k act as calcium transporter in intestine. When DPs and VSEE was added, the expression of calbindinD9k decreased and did not show any significant difference (*p* > 0.05) compared to the non-injected group. This reveals that DPs and VSEE could promote calcium uptake by acting as calcium carriers. The significant increase in the expression of Zip4 and SI (*p* < 0.05) in the lentil treatment group indicates that intra-amniotic administration of lentil improved BBM functionality. As is evident in the current study, it suggested that dietary prebiotics might lead to enterocyte proliferation and BBM functionality. However, this effect was not observed in DPs and VSEE treatment groups.

Overall, the increase in the relative abundance of beneficial probiotics, intestinal villus surface area, and goblet cell diameters, in addition to the change of calcium-related gene expression, suggest that chickpea prebiotic, lentil prebiotic and DPs are promising in improving Ca status. Prebiotics extracts from chickpea and lentil improve calcium bioavailability by promotion of gut beneficial bacterial populations levels, the enlargement of gut villus surface area and the improvement of functional activity. Duck egg white peptides and VSEE promote calcium uptake through the reaction with calcium, acting as calcium carriers and maintaining gut health.

## 5. Conclusions

This study demonstrated that the intra-amniotic administration of chickpea and lentil prebiotics extracts and duck egg white peptides may improve calcium bioavailability by the promotion of beneficial bacterial populations (*Bifidobacterium* and *Lactobacillus*) and limitation of potentially pathogenic bacterial populations. Moreover, the expression of BBM functional genes was affected by lentil prebiotics extract. DPs and VSEE may promote and contribute to calcium uptake. Additional research is needed to further establish the specific relationship and potential synergistic effects between chickpea and lentil prebiotics, or duck egg white peptides on gut health, effects on specific bacterial species, and BBM calcium transport pathways.

## Figures and Tables

**Table 1:** DNA sequences of the primers used in this study.

Analyte	Forward Primer (5′-3′) (Nucleotide Position)	Reverse Primer (5′-3′)	Base Pair	GI Identifier
Zip4	TCTCCTTAGCAGACAATTGAG	GTGACAAACAAGTAGGCGAAAC	95	107050877
DMT-1	TTGATTCAGAGCCTCCCATTAG	GCGAGGAGTAGGCTTGTATTT	101	206597489
CalBindinD9k	CCTGCAGAAATGAAGAGCATTTT	CTCCATCGCCATTCTTATCCA	125	SAMN06269460
CalBindinD28k	CGGAGGAGAATTTCCTGTTGT	CCACTGTGGTCACTGTCATATT	97	NM_205513.1
PMCA1b	TGCAGATGCTGTGGGTAAAT	CCATAAGGCTTCCGCAATAGA	100	NM_001168002.3
NCX1	CCTGACGGAGAAATAAGGAAGA	CCCAGGAGAAGACACAGATAAA	114	NM_001079473.1
SPP1	GACACTGACGAGTCTGATGAAG	CTGAAGCCATATGCCACACT	113	NM_204535.4
SI	CCAGCAATGCCAGCATATTG	CGGTTTCTCCTTACCACTTCTT	95	2246388
TRPV6	GCTCCCAGAACCTTCTCTATTT	CCAGGTAATCCTGAGCTCTAATG	123	XM_004938142.2
AP	CGTCAGCCAGTTTGACTATGTA	CTCTCAAAGAAGCTGAGGATGG	138	45382360
AKP	CTCATTCCAGCGTACTCTTCTT	GTGTGTAGATCAAAGGGCTACT	100	XM_015291489.1
18S rRNA	GCAAGACGAACTAAAGCGAAAG	TCGGAACTACGACGGTATCT	100	7262899

Zip4, Zinc transporter 4; DMT1, Divalent metal transporter 1; CalBindinD9k, Ca transporter D9k; CalBindinD28k, Ca transporter D28k; PMCA1b, Plasma membrane Ca^2+^ ATPase; NCX1, Sodium calcium exchanger 1; SPP1, Secreted Phosphoprotein 1; SI, Sucrose isomaltase; TRPV6, Transient receptor potential vanilloid 6calcium channel; AP, Amino peptidase; AKP, Alkaline Phosphatase; 18S rRNA, 18S Ribosomal subunit.

**Table 2:** Effect of chickpea prebiotics, lentil prebiotics, DPs and VSEE on the Bone and liver mineral content.

Group	Bone (mg/kg)				Liver (mg/kg)			
	Ca	Mg	Fe	Zn	Ca	Mg	Fe	Zn
Non-injected	22,985 ± 962.5	370 ± 8.72	12.8 ± 0.56	31.4 ± 2.08	56.4 ± 1.89	165.4 ± 4.05	55.4 ± 5.89	23.0 ± 1.35
18 MΩ H_2_O	21,253 ± 431.6	339 ± 5.56	12.4 ± 1.12	33.9 ± 1.54	56.2 ± 1.83	157.7 ± 5.80	43.6 ± 4.63	22.2 ± 1.18
Ca	25,173 ± 1552.6	364 ± 18.30	12.1 ± 0.99	34.1 ± 0.94	51.1 ± 1.96	158.0 ± 2.90	48.3 ± 3.53	22.1 ± 1.66
Chickpea + Ca	23,271 ± 1172.5	345 ± 5.14	10.8 ± 0.92	35.9 ± 1.72	55.7 ± 2.03	152.5 ± 3.77	52.6 ± 3.81	24.2 ± 3.23
Lentil + Ca	23,823 ± 1123.7	397 ± 24.36	11.9 ± 0.80	33.4 ± 1.32	55.7 ± 2.77	166.5 ± 4.34	51.9 ± 6.94	24.5 ± 2.84
DPs + Ca	25,981 ± 2518.5	335 ± 54.94	11.0 ± 0.72	34.9 ± 2.55	55.3 ± 2.73	158.1 ± 4.44	53.2 ± 7.43	24.4 ± 2.36
VSEE + Ca	22,587 ± 1046.2	350 ± 13.96	13.6 ± 1.37	32.8 ± 0.87	64.9 ± 2.74	161.6 ± 3.39	60.5 ± 4.98	25.1 ± 2.90
Chickpea	24,910 ± 1397.2	374 ± 15.14	13.7 ± 0.93	33.2 ± 3.06	63.6 ± 3.68	164.9 ± 4.68	51.5 ± 5.59	20.8 ± 1.76
Lentil	24,824 ± 1178.7	394 ± 8.84	13.0 ± 1.48	35.9 ± 2.13	66.7 ± 2.44	164.5 ± 2.75	50.6 ± 7.55	24.1 ± 1.67
DPs	24,967 ± 1849.3	381 ± 33.63	11.2 ± 0.95	30.4 ± 0.63	60.8 ± 4.99	156.2 ± 2.98	55.3 ± 5.47	26.5 ± 2.38
VSEE	24,582 ± 655.4	368 ± 10.98	13.9 ± 0.77	30.8 ± 0.75	61.8 ± 1.60	164.9 ± 3.69	60.0 ± 7.26	22.8 ± 1.26

DPs, Duck Egg white Peptides; VSEE, Val- Ser-Glu-Glu.

## Abbreviations

Ca	Calcium
Fe	Iron
Mg	Magnesium
Zn	Zinc
DPs	Desalted duck egg white peptides
VSEE	Val-Ser-Glu-Glu
CPPs	Casein phosphopeptides
TRPV6	Transient receptor potential vanilloid 6
PMCA1b	Plasma membrane Ca^2+^ ATPase
SPP1	Secreted Phosphoprotein 1
NCX1	Sodium calcium exchanger 1
DMT1	Divalent metal transporter 1
AP	Amino peptidase
SI	Sucrose isomaltase
BBM	Brush border membrane
